# Numerical Strength Analysis of Laser-Welded Differential Housing and Gear Considering Residual Stress

**DOI:** 10.3390/ma16134721

**Published:** 2023-06-29

**Authors:** Liuping Wang, Zhengshun Ni, Yingang Xiao, Yongqiang Li, Xianghuan Liu, Yongzhi Chen, Shuanghao Cui, Dejun Zhang, Chengji Mi, Quanguo He

**Affiliations:** 1 College of Mechanical Engineering, Hunan University of Technology, Zhuzhou 412007, China; wangliuping_hut@126.com (L.W.); nizhengshun@126.com (Z.N.); xiaoyingang_hut@126.com (Y.X.); liyongqiang_hut@126.com (Y.L.); hequanguo@hut.edu.cn (Q.H.); 2 Laser Welding Department, Zhuzhou Gear Co., Ltd., Zhuzhou 412007, China; liuxianghuan_zg@126.com (X.L.); chenyongzhi_zg@126.com (Y.C.); cuishuanghao_zg@126.com (S.C.); zhangdejun_zg@126.com (D.Z.)

**Keywords:** laser welding, residual stress, strength analysis, differential housing

## Abstract

In order to avoid slackening of differential housing and gear joined by bolts, the laser-welding process is proposed in this paper, and the strength of a connecting joint was estimated by numerical analysis with consideration of welding residual stress. The process parameters of laser welding for dissimilar materials QT600 cast iron and 20MnCr5 structural alloy steel were introduced, and chemical composition analysis and microstructure analysis were conducted on the welded joints. The finite element model of laser-welded differential housing and gear was established to obtain the welding residual stress by applying a moving heat source. To verify the accuracy of the simulated result, static pressing tests were employed. The maximum tensile residual stress was 319.4 MPa, located at the same point as the maximum temperature. The simulated stress agreed well with the experimental data. Finally, the dynamic strength of laser-welded differential housing and gear under forward, reverse, and start-up conditions was assessed by regarding welding residual stress as the initial stress field, which showed that all safety factors were greater than 1.4.

## 1. Introduction

The reliability of differential used in cars plays an important part in its safety and quality. Traditionally, differential housing and the gear are connected by bolts, which may generate looseness after servicing a long time. In order to ensure the reliability of the differential, the laser-welding process is presented to replace the traditional connecting type. Because of lower melting depth and a smaller heat-affected zone with laser welding, laser-welded differential housing and gear become possible [1,2,3,4]. However, it inevitably generates residual stress, and the strength of welded joints from dissimilar 20MnCr5 and QT600 materials is of concern [5]. Differential housing is cast from QT600, and the gear is milled from 20MnCr5.

The laser-welding process produces fusion between differential housing and a gear through heat input [6,7,8]. If the laser-welding parameters are reasonably controlled, the metallurgical interaction between the added wire and the base metal can effectively reduce the tendency of pre-cracks, enhance mechanical properties of the weldment, and suppress softening and embrittlement of the welded joints. Additionally, the highly concentrated energy density in this process could result in low welding-heat input and minimal post-weld residual deformation [9,10]. However, during the cooling period, residual stress is formed [11,12,13]. In order to accurately estimate the strength of laser-welded joints, a quantitative characterization of welding residual stress is needed [14].

Currently, welding residual stress is mainly determined by a numerical simulation. The technique of birth–death elements was employed to simulate the welding process and investigate the patterns of the temperature field and stress field of dissimilar metals [15]. A study on the effects of multi-pass welding and post-weld heat treatment has been conducted with welding of dissimilar metal pipe joints [16]. A simulation of the electron beam welding of titanium–steel dissimilar metals has been created, and the influence of the filler metal on the strength assessment was discussed [17]. With the molten pool considered as a kind of laminar flow, a copper–nickel dissimilar-metal laser-welding process has been simulated [18]. The characteristics of the molten pool and weld microstructure of copper–aluminum dissimilar metal connected by laser welding have been studied, and the influence of welding speed and power on strength of welded joints has been discussed [19]. However, dissimilar-metal laser welding between cast iron and steel has not been extensively investigated.

The laser-welding parameters of 20MnCr5 and QT600 dissimilar materials were firstly displayed, and the chemical compositions as well as microstructure of welded joints were analyzed. Then, the residual stress of laser-welded differential housing and gear was simulated, the numerical calculated results were compared with the experimental data. The strength of the laser-welded differential housing and gear under three extreme conditions was estimated.

## 2. Experiment

### 2.1. Laser-Welding Parameters and Preparation of Specimen

**Figure 1:**
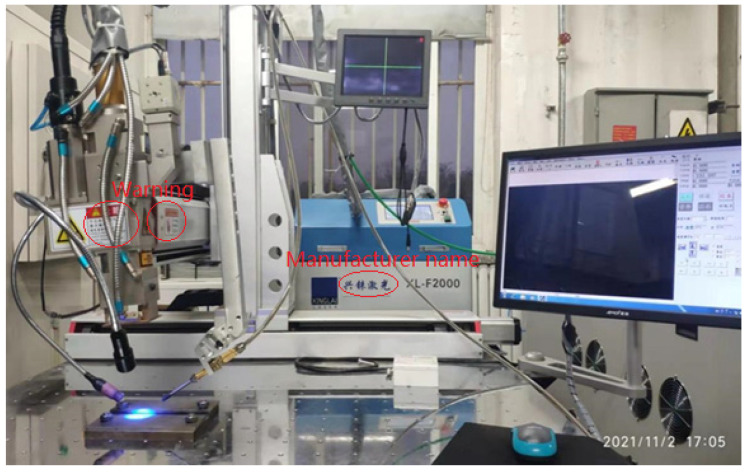
Semiconductor laser-welding equipment.

The laser welding for 20MnCr5 and QT600 dissimilar materials was carried out using an XL-F2000 stationary semiconductor laser-welding machine (made by Xinglin Laser-welding Co. Ltd., Liaocheng City, China), as shown in Figure 1. A welding wire with a diameter of ø1 mm was needed. The chemical compositions of the base metals and welding wire are presented in Table 1. Two plates with 50 mm length, 15 mm width, and 2 mm thickness were butt-welded. The connecting part had a V-shaped groove, and the slope angle on each side was 10°. The laser power was 1500 w, and the defocus was −2 mm. The range of welding speed was from 0.3 m/min to 0.4 m/min, while the wire-feeding speed was 1.0 m/min and the wire-feeding angle was 15° of automatic wire feeding. The room temperature was 20 °C.

**Figure 2:**
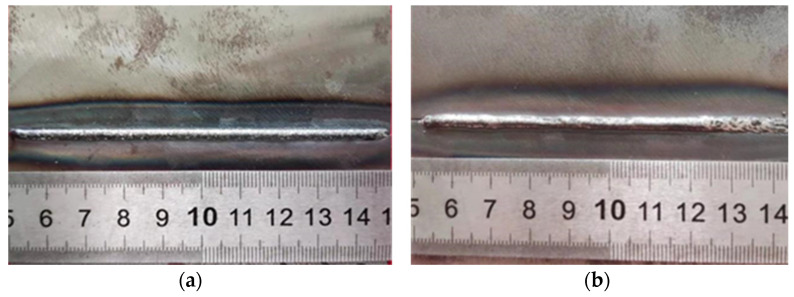
Laser-welding specimen: (**a**) top surface, (**b**) bottom surface.

The top and bottom surfaces of laser-welded joints are shown in Figure 2. When the heat source from the laser head melted the materials on the top side, the surface on the other side was free to form the seam weld as well. The continuously smooth seam weld on both sides is clearly seen in Figure 2, and the welding defects such as undercut, porosity, or cracks were barely observed. A small number of defects appeared due to uncertain factors of the welding process. Only a slight spattering of small particles attended around the welding region, but it did not affect the performance of the welded joints. The surface of the seam weld did not show significant oxidation, even though the protective gas was not used.

### 2.2. Elements Distribution and Microstructure

**Figure 3:**
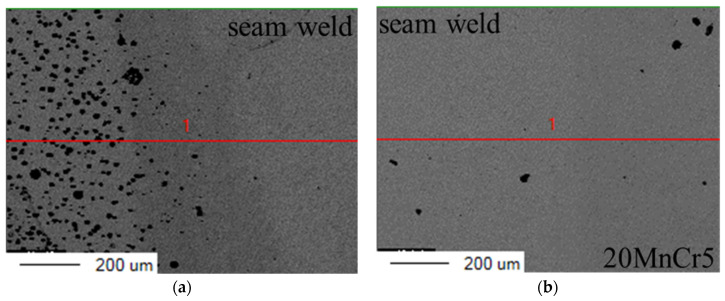
Route between different regions: (**a**) QT600 and seam weld, (**b**) 20MnCr5 and seam weld.

**Figure 4:**
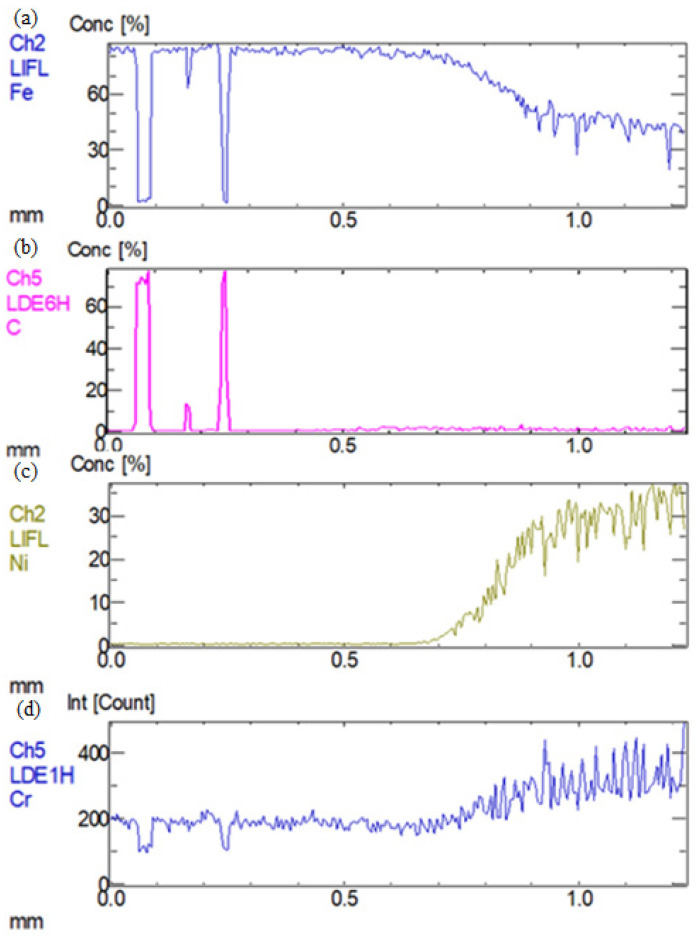
Elements’ distribution in interface region between QT600 and seam weld: (**a**) Fe, (**b**) C. (**c**) Ni, (**d**) Cr.

**Figure 5:**
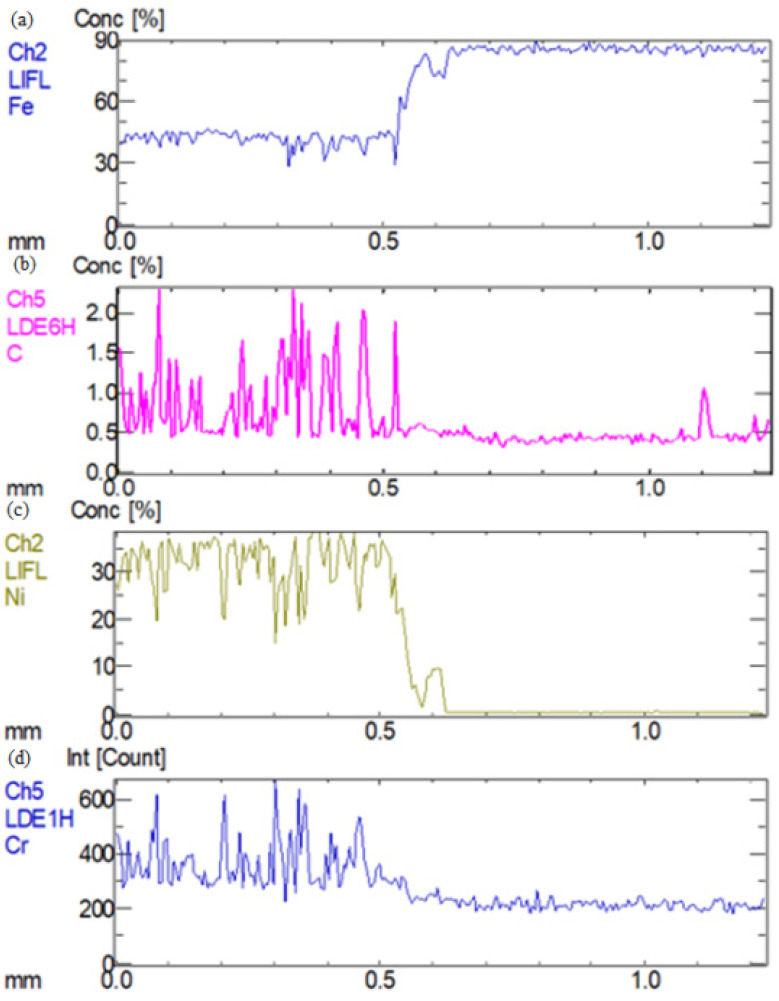
Elements’ distribution in interface region between 20MnCr5 and seam weld: (**a**) Fe, (**b**) C, (**c**) Ni, (**d**) Cr.

In order to analyze the elements distribution along the heat-affected zone of welded joints a route was chosen, displayed in Figure 3, and the measuring distance was 1 mm. The route was located at the interface area between the base metals and the seam weld. The distribution of Fe, C, Ni, and Cr elements in the interface region was obtained by X-ray diffraction and is shown in Figure 4 and Figure 5. It could be clearly seen that Fe and C elements had no significant offset in the interface region between QT600 and the seam weld, but it was the opposite for the other two elements. The range of the transition region for Ni and Cr along the selected route was greater than that for other elements. In Figure 5, C only precipitated in small amounts at some regions. Fe and Ni exhibited gradient variation characteristics. Cr behaved stably in the base metal, but it presented a certain fluctuation state in the seam weld.

**Figure 6:**
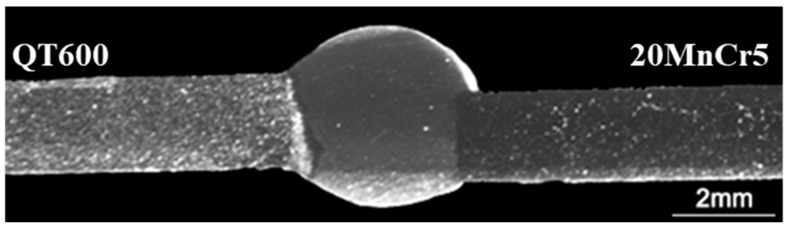
Longitudinal section of welded joint investigated by a microscope.

**Figure 7:**
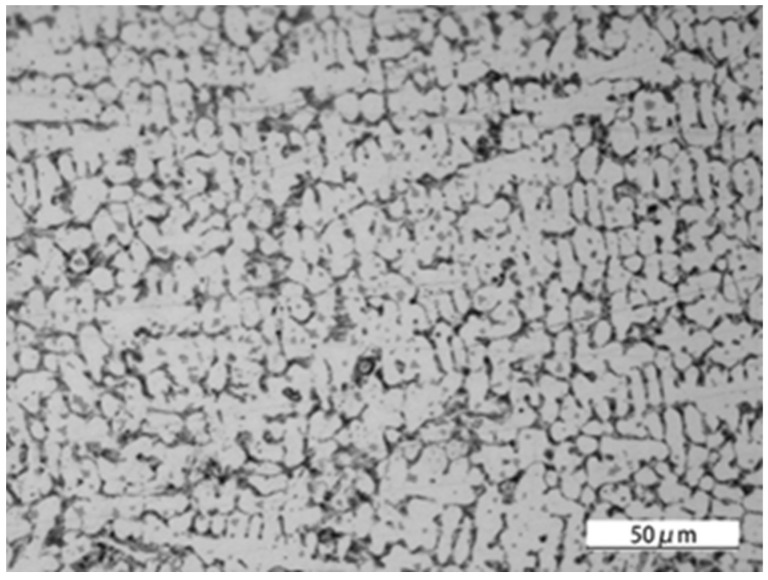
Microstructure in the seam weld.

**Figure 8:**
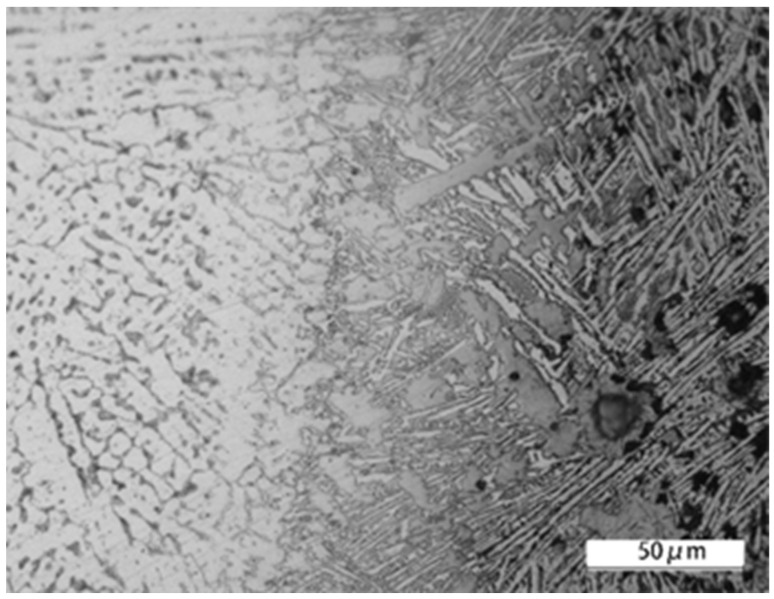
Microstructure around the interface between QT600 and seam weld.

**Figure 9:**
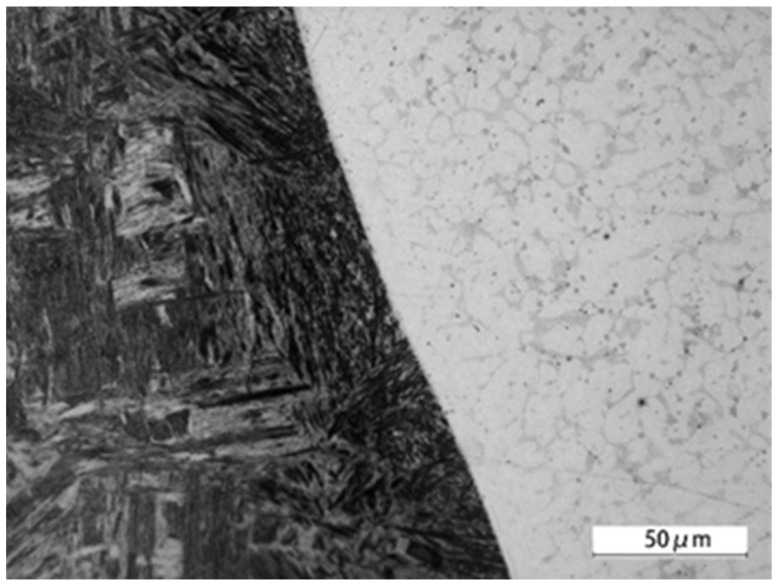
Microstructure around the interface between 20MnCr5 and seam weld.

A microscopic structure analysis was conducted on the seam weld obtained from the laser-welding process, using an optical microscope. The longitudinal section of the welded joint is shown in Figure 6, and defects were not seen in the connection part. The microstructure in the region of the seam weld was observed with a scanning electron microscope, and is displayed in Figure 7. The porosity or crack defects were not found. The insufficient dilution observed in the welding process was a result of intentionally reducing the dilution ratio, aimed at improving welding efficiency and reducing heat input and the heat-affected zone to mitigate welding deformation and residual stress, and thereby it enhanced dimensional welding accuracy. It could be clearly seen that a mixed structure of dendrites and equiaxed crystals was formed. The microstructure around the interface between QT600 and seam weld is shown in Figure 8. A transition zone with a thickness of approximately 100 μm was observed, and some martensite and ledeburite structures were formed. The microstructure around the interface between 20MnCr5 and the seam weld is displayed in Figure 9. The interface between 20MnCr5 and the seam weld was continuous and smooth, and the crystal microstructure was complete, forming a single-phase austenite organization.

**Figure 10:**
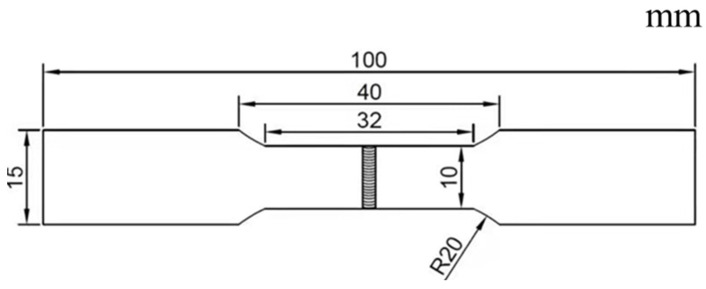
Schematic diagram of the tensile specimen.

Mechanical performance tests were conducted on the laser-welded specimens, wherein the tensile strength of the base metal and the welded joint were compared using a PLD-100 electric hydraulic servo universal testing machine (made by Lichuang Testing Co., Ltd., Xian City, China). The welding joint specimens used in the experiment were dog-bone-shaped specimens, and the sampling method for the specimens was prepared in accordance with the ISO 4136-2001 standard [20]. The dimensions of the tensile specimen are shown in Figure 10. The tensile strength of base metal QT600 obtained from three sets of tensile tests were 550, 554, and 560 MPa, while the tensile strength of the welded joints was 480, 483, 485 MPa, accounting for 88.2% of that of the base metal QT600. The failure location of the testing specimen was located at the root of the weld toe.

## 3. Simulation of Residual Stress

### 3.1. Geometric Modeling

**Figure 11:**
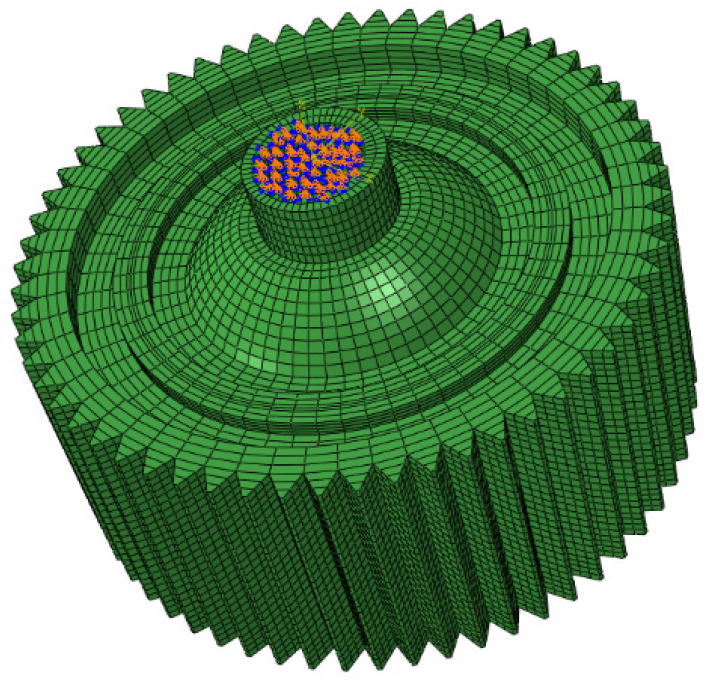
Finite element model of laser-welded differential housing and gear.

In order to estimate the strength of laser-welded differential housing and gear under different conditions, a finite element model was firstly built to obtain the welding residual stress through a simulation of the welding process. Some details of chamfer and hole were simplified in the model. In order to improve computational efficiency, the mesh was refined in the welded zone, while a relatively coarse mesh was utilized in other areas. The mesh model that consisted of 40,980 nodes and 28,860 solid elements is shown in Figure 11. ABAQUS v14.0 software was utilized to simulate the fully coupled thermal analysis, including the welding process and cooling period. Based on the UMAT programming language, a moving heat source was applied to simulate the welding process. Then, a thermal-structural coupling analysis was performed to obtain the welding residual stress. Before applying the moving heat resource, the end of differential housing was constrained by all degrees of freedom, as shown in Figure 11. In order to save time in a static-pressing test, one-sixth length seam weld along the circle was created, as well as another one-sixth length seam weld in its symmetrical location. Therefore, in the following simulation, the same seam weld was considered to obtain welding residual stress and analyze dynamic strength.

### 3.2. Heat Source Model

To characterize a real heat source, dual-ellipsoidal heat sources were selected to simulate the actual welding process [21,22]. The dual-ellipsoidal volume heat source was divided into two parts, and the distribution of heat flux density was a function of the thermal inputs of the front and rear semi-ellipsoidal bodies. The heat flow distribution in the front half ellipsoid was described as:

(1) $$\begin{array}{l} q_{1}\left(x,y,z\right)=\frac{6\sqrt{3}\left(f_{1}Q\right)}{a_{1}bc\pi \sqrt{\pi }}\\ \exp\left(-\frac{3x^{2}}{a_{1}^{2}}-\frac{3y^{2}}{b^{2}}-\frac{3z^{2}}{c^{2}}\right),x\geq 0 \end{array}$$

where Q is heat inputs, $a_{1},b,c$ is the axis of front half ellipsoid along the x,y,z directions, $f_{1}$ is the ratio of heat input in the front half ellipsoid.

Then, the heat flow distribution in the rear half ellipsoid was expressed as:

(2) $$\begin{array}{l} q_{2}\left(x,y,z\right)=\frac{6\sqrt{3}\left(f_{2}Q\right)}{a_{2}bc\pi \sqrt{\pi }}\\ \exp\left(-\frac{3x^{2}}{a_{2}^{2}}-\frac{3y^{2}}{b^{2}}-\frac{3z^{2}}{c^{2}}\right),x<0 \end{array}$$

where $a_{2}$ is the axis of the rear half ellipsoid along the x direction, and $f_{2}$ is the ratio of heat input in rear half ellipsoid.

The relationship between $f_{1}$ and $f_{2}$ could be calculated as:

(3) $$f_{1}+f_{2}=2$$

(4) $$f_{1}=\frac{2a_{1}}{a_{1}+a_{2}}$$

(5) $$f_{2}=\frac{2a_{2}}{a_{2}+a_{1}}$$

### 3.3. Boundary Conditions

In the process of thermal coupling calculation, the fundamental modes of heat transfer include heat conduction, convective heat transfer, and radiative heat transfer. Heat conduction can be determined as:

(6) $$\rho C\frac{\partial T}{\partial t}\left(x,y,z,t\right)=-\nabla q\left(x,y,z,t\right)+Q\left(x,y,z,t\right)$$

where ρ is density, C is specific heat, and t is time.

Then, Fourier heat flux could be described as:

(7) q=−k∇T

where k is thermal conductivity and T is temperature.

Heat exchange between the welded component and the surrounding air occurrs primarily through convective heat transfer and radiative heat transfer. The calculation of convective heat transfer was based on the law of convection:

(8) $$q_{c}=a_{c}\left(T-T_{0}\right)$$

where $a_{c}$ is the convective heat transfer coefficient, 20 W/m^2^·K, and $T_{0}$ is ambient temperature, 25 °C in this paper.

The calculation of radiative heat transfer was based on the Stefan–Boltzmann law:

(9) $$q_{r}=-\varepsilon \sigma \left[{\left(T-T_{f}\right)}^{4}-{\left(T_{0}-T_{f}\right)}^{4}\right]$$

where σ is the radiation constant, 5.67 × 10 W/(m^2^·K^4^), ε is the blackness coefficient, and $T_{f}$ is absolute zero.

### 3.4. Coordinate Transformation

In order to simulate a circular moving heat source, a coordinate transformation was presented in this paper. If the welding process was clockwise, the position could be expressed as:

(10) $$X_{1}=x_{0}+r\left(1-\cos\theta \right)$$

(11) $$Y_{1}=y_{0}+r\sin\theta$$

where $X_{1}$ and $Y_{1}$ are the end coordinate values in x and y directions, $x_{0}$ and $y_{0}$ are the starting coordinate values in x and y directions, θ is the center angle of the circle, and r is the radius of the circle, provided by the model dimensions of the differential gear shaft as 60 mm.

When the welding process was counterclockwise, the position was determined as:

(12) $$X_{2}=x_{0}+r\sin\theta$$

(13) $$Y_{2}=y_{0}+r(1-\cos\theta )$$

where $X_{2}$ and $Y_{2}$ are the end coordinate values in x and y directions.

In addition, welding arc length l could be expressed as:

(14) l=vt

where v is the welding peripheral speed.

The center angle of the circle θ can be described as:

(15) θ=l/r

### 3.5. Simulation of Welding and Static-Pressing Process

Based on the equations mentioned above, the parameters used in this paper are listed in Table 2. The formation of residual stress included two parts, welding process and cooling period, so cooling time $t_{c}$ is also listed in Table 2. The welding peripheral speed was 0.02 m/s.

**Figure 12:**
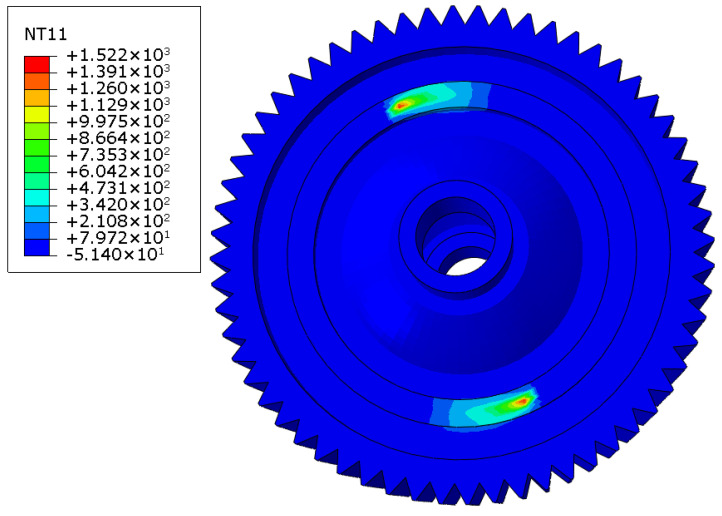
Temperature field (unit: °C).

**Figure 13:**
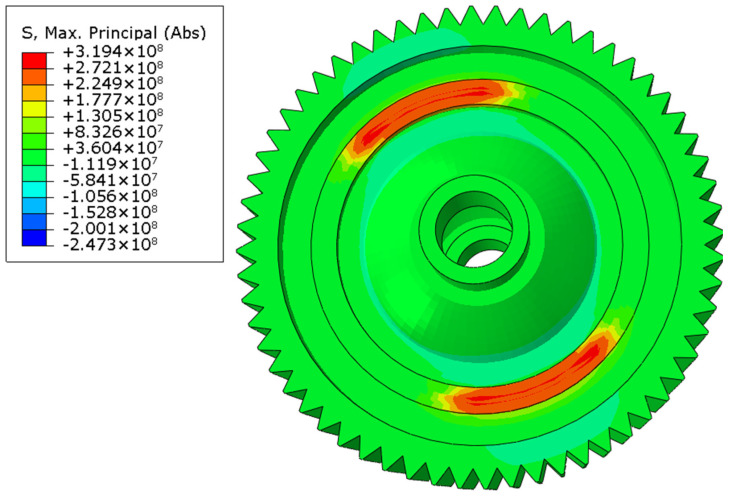
Welding residual stress field (unit: MPa).

**Figure 14:**
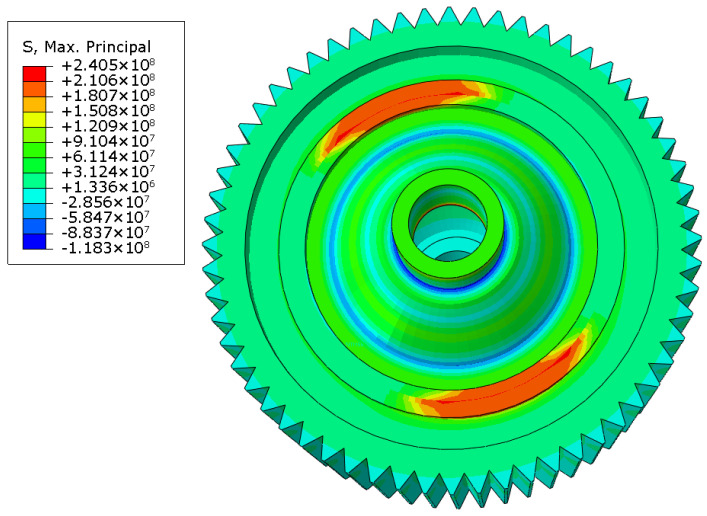
Principal stress field (unit: MPa).

According to the input parameters listed in Table 2, the temperature field of laser-welded differential housing and gear after the seam weld in one circle was finished is shown in Figure 12. The maximum temperature was located at the end point of laser welding and reached up to 1522 °C. Because the two symmetrical one-sixth length seam welds simultaneously welded, the temperature distribution was also symmetrical. After the welding process was finished, the welded structure was cooled to room temperature in a period of 3000 s. When the cooling process was completed, the welding residual stress was formed, as shown in Figure 13. The maximum tensile residual stress was 319.4 MPa, located at the same point as the maximum temperature. In order to compare the stress field with the static pressing test, one end face of laser-welded differential housing and gear was constrained by all degrees of freedom, and 10 tons of pressure was applied to the end face of differential housing on the other side. Before running this simulation, the calculated welding residual stress was considered as the initial stress field. Then, the distribution of the principal stress field was obtained, as shown in Figure 14. The maximum stress was 240.5 MPa, located at the end point of the seam weld. The high-stress area covered the entire seam weld.

**Figure 15:**
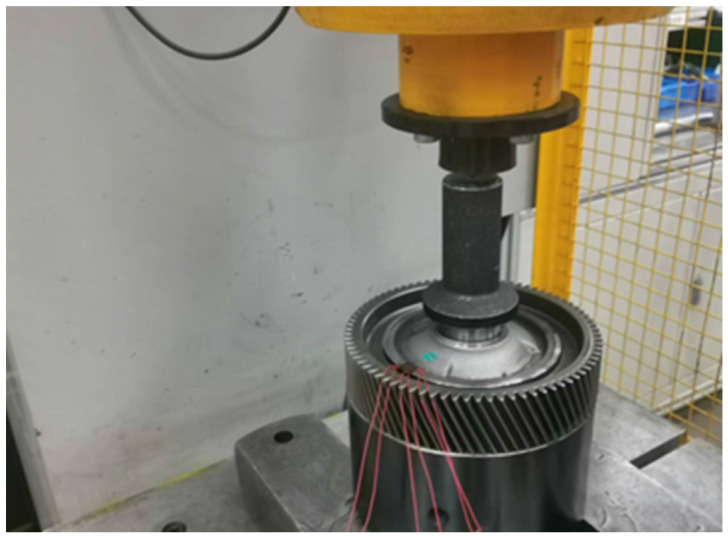
Static-pressing test.

**Figure 16:**
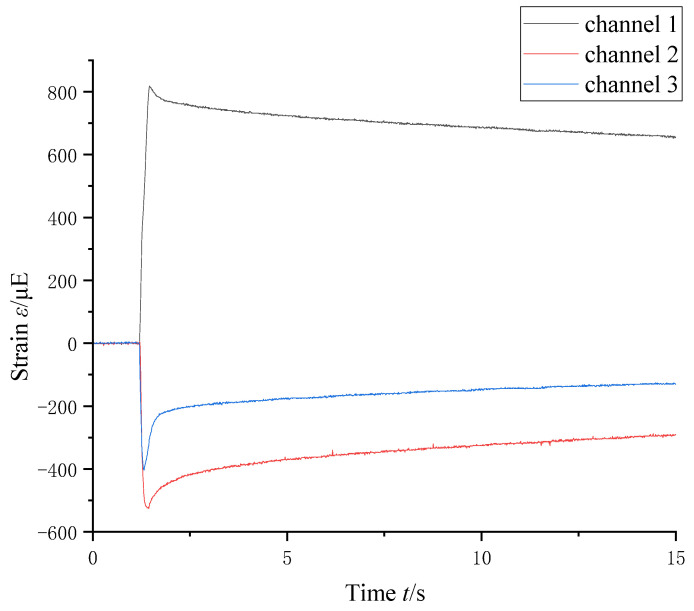
Strain history of welded joint.

In order to assess the mechanical performance of laser-welded differential housing and gear, a static-pressing test was conducted on a physical prototype, as shown in Figure 15. At the same time, the strain history located at the maximum stress point was measured by the strain rosette, as shown in Figure 16. When Young’s modulus of the welded joint was 205 GPa, the measuring stress was 220.7 MPa. The relative error between the simulated result and the experimental data was only 8.9%, which meant that the presented simulation method was correct and reliable.

## 4. Dynamic Strength Estimation

In this paper, in order to estimate the dynamic strength estimation under three typical conditions of forward running, reverse running, and start-up, a finite element analysis of laser-welded differential housing and gear was conducted with consideration of welding residual stress. The end of differential housing was constrained by all degrees of freedom, while one tooth surface was applied by the loads produced from the resistance. The loads in three directions could be described as [23]:

(16) $$\left\{ \begin{array}{l} F_{t}=2T_{d}/d\\ F_{r}=F_{t}\tan\alpha_{n}/\cos\beta \\ F_{a}=F_{t}\tan\beta \end{array}\right.$$

where $F_{t}$, $F_{r}$, and $F_{a}$ are the circumferential, radial, and axial forces, respectively. $T_{d}$ is the torque transmitted by the gear, d is the diameter of the gear-indexing circle, $\alpha_{n}$ is the normal pressure angle, equal to 20°, β is the helix angle of the indexing circle, equal to 8°. The forces in three conditions when the maximum torque of the differential gear was 1800 N·m are listed in Table 3.

**Figure 17:**
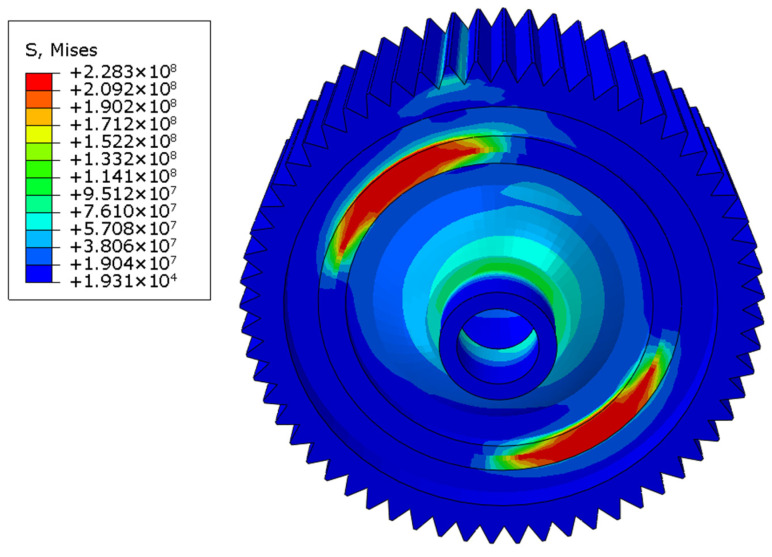
von Mises stress field under forward running.

**Figure 18:**
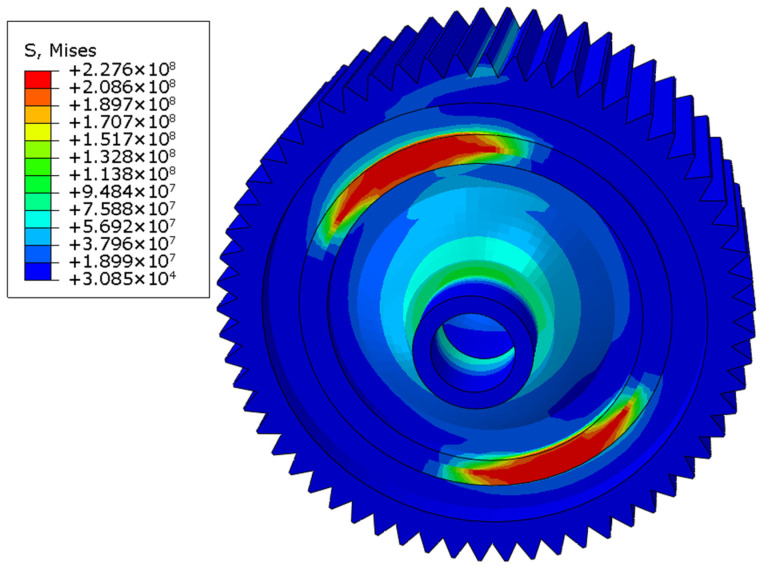
von Mises stress field under reverse running.

**Figure 19:**
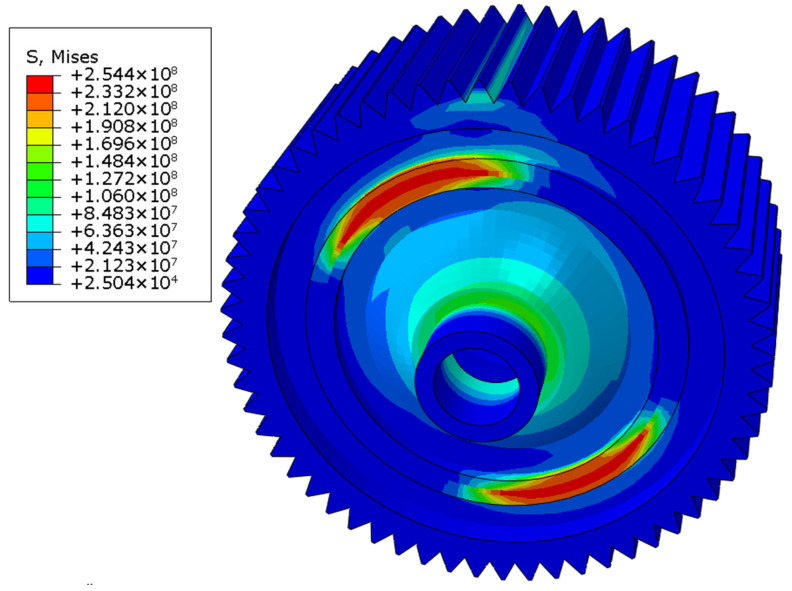
von Mises stress field under start-up condition.

Then, similarly, welding residual stress was considered as the initial stress field, and the loads were applied at the reference point, which was on the top of one gear surface and was coupled with the nodes of that gear surface. The distributions of the stress field in three working conditions are shown in Figure 17, Figure 18 and Figure 19. The maximum stress was 228.3 MPa in the forward-running condition and was located at the laser-welding seam, where the high-stress region appeared. The stress concentration zone was also found in the root of the gear, and the stress level was around 180 MPa. In the other two cases, the stress distribution was similar, while the maximum stress was 227.6 and 254.4 MPa, respectively. Based on the tensile test, the yield limit of the laser-welded joint was 370 MPa, so the dynamic strength with laser welding of differential housing and gear met the design requirement. The safety factor under three typical conditions of forward running, reverse running, and start-up was 1.62, 1.63, and 1.45, respectively.

## 5. Conclusions

In this paper, a laser-welding process was presented to replace the threaded connection for differential housing and gear. The welding technology parameters for dissimilar materials QT600 cast iron and 20MnCr5 structural alloy steel were firstly introduced, and the chemical compositions and the distribution of elements along the interface route were shown. The microstructure of the welded joint was observed, and the welded seam had no holes, cracks, or other defects. The tensile strength of the welded joint reached up to 88.2% of the strength limit of the base metal QT600. Then, the welding process was simulated to obtain welding residual stress by thermal-structural analysis. The maximum tensile residual stress was 319.4 MPa, located at the same point as the maximum temperature. In consideration of welding residual stress, the stress filed under 10 tons of static pressing was obtained. The simulated results agreed well with the experimental data. Furthermore, the dynamic strength of laser-welded differential housing and gear was assessed by regarding welding residual stress as the initial stress field, and its safety factor under three typical conditions of forward running, reverse running, and start-up was 1.62, 1.63, and 1.45, respectively.

## Figures and Tables

**Table 1:** Main chemical composition (%).

	C	Si	Mn	P	S	Cr	Mg	Ni	Fe
OT600	3.6–3.8	2.4–2.8	0.3–0.5	≤0.1	≤0.035	-	0.04–0.05	-	Bal
20MnCr5	0.17–0.22	≤0.25	1.1–1.5	≤0.035	≤0.035	1–1.3	≤0.15	-	Bal
Weld wire	≤0.1	≤0.5	2.5–3.5	≤0.04	≤0.04	18–22	-	≥67	≤3.0

**Table 2:** Input parameters.

$t_{c}$/s	Q/J	$a_{1}$/mm	$a_{2}$/mm	b/mm	c/mm	ρ/kg/m^3^	C J/(kg °C)	k W/(m·K)	ε
3000	1200	0.003	0.006	0.003	0.003	7600	450	35	0.8

**Table 3:** Forces on tooth surface under three working conditions.

	$F_{t}$/N	$F_{r}$/N	$F_{a}$/N
Forward running	19,565	−7191	2749.7
Reverse running	−19,565	−7191	−2749.7
Start-up condition	25,434	−9360	3561

## Data Availability

Not applicable.
